# Tips for percutaneous nephrolithotomy for transplant kidney stone

**DOI:** 10.1002/iju5.12800

**Published:** 2024-10-13

**Authors:** Takafumi Yagisawa, Tomokazu Shimizu, Ayane Tachiki, Yudai Ishiyama, Tadashi Onohara, Shoichi Iida, Hideki Ishida, Toshio Takagi

**Affiliations:** ^1^ Department of Urology and Kidney Transplantation Toda Chuo General Hospital Saitama Japan; ^2^ Department of Urology Tokyo Women's Medical University Tokyo Japan; ^3^ Department of Organ Transplant Medicine Tokyo Women's Medical University Tokyo Japan

**Keywords:** allograft, kidney calculi, kidney transplantation, lithotripsy, percutaneous nephrolithotomy

## Abstract

**Introduction:**

The management of urinary tract stones, particularly *de novo* kidney allograft stones, presents unique challenges for kidney transplant recipients because of their prevalence and specific clinical considerations. Here, we describe a case in which percutaneous nephrolithotomy was successfully used to fragment a large kidney allograft stone ≥20 mm in size.

**Case presentation:**

A 57‐year‐old woman who underwent ureteroureterostomy post simultaneous pancreas–kidney transplantation presented with gross hematuria after 15 years. Computed tomography revealed a 23‐mm stone in the transplanted kidney. Initial attempts at endoscopic combined intrarenal surgery were changed to percutaneous nephrolithotomy because of poor ureter mobility and tortuosity. Stone fragmentation was achieved using pneumatic and ultrasonic lithotripsy. A second procedure using Swiss LithoClast^®^ Trilogy enabled complete stone clearance and ureteral stent placement.

**Conclusion:**

By understanding the peculiarities of the percutaneous approach, we demonstrated the safe and effective use of a pneumatic and ultrasonic lithotripter for kidney allograft stone fragmentation.

**Keynote message**

The key message of this report is the management of *de novo* kidney allograft stones using endoscopic lithotripsy techniques, such as percutaneous nephrolithotomy. Despite the challenges posed by the unique anatomy and clinical features of kidney transplant recipients, we demonstrate the safety and efficacy of percutaneous nephrolithotomy with pneumatic and ultrasonic lithotripsy in achieving stone fragmentation in the reported case. Our findings emphasize the importance of individualized surgical strategies and close perioperative management for optimizing clinical outcomes for kidney transplant recipients with kidney stones.

**Abbreviations & Acronyms**
CT: computed tomography
IRP: intrarenal pressure
PCNL: percutaneous nephrolithotomy
TUL: transurethral lithotripsy

## Introduction

Urinary tract stones, also known as *de novo* kidney allograft stones, occur specifically in kidney allografts after transplantation. They have a frequency ranging from 0.2% to 2.0%, indicating a low prevalence.^1^, ^2^, ^3^ Owing to the solitary kidney status and immunosuppression in kidney transplant recipients, there is a heightened risk of acute renal failure or severe urinary tract infections resulting from urolithiasis.^4^ Additionally, the non‐physiological urinary tract anatomy after transplantation can be challenging for performing interventions such as ureteral stenting or endoscopic approaches. While renal stones are often be treated with TUL, PCNL for stone fragmentation may be preferred in cases involving transplanted kidneys. However, there are a limited number of reports on PCNL for *de novo* kidney allograft ureteral stones. Here, we report our experience using a percutaneous approach and a device integrating pneumatic and ultrasonic lithotripsy for PCNL in a case of a *de novo* kidney allograft stone.

## Case presentation

**Fig. 1:**
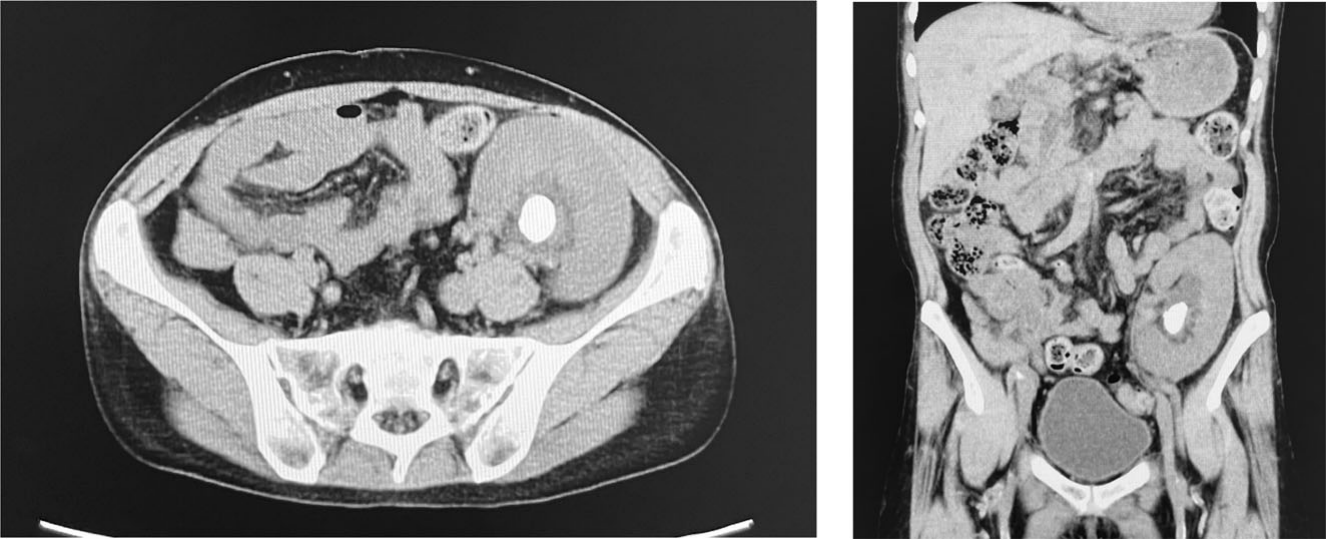
Preoperative CT images of a kidney allograft stone. The present case pertained to a simultaneous pancreas–kidney transplant recipient, with the transplanted kidney placed in the left iliac fossa. The transplanted renal pelvis showed a stone measuring 23 mm in its longest diameter, with an average CT value of 856 Hounsfield Units (maximum, 1092; minimum, 504).

**Fig. 2:**
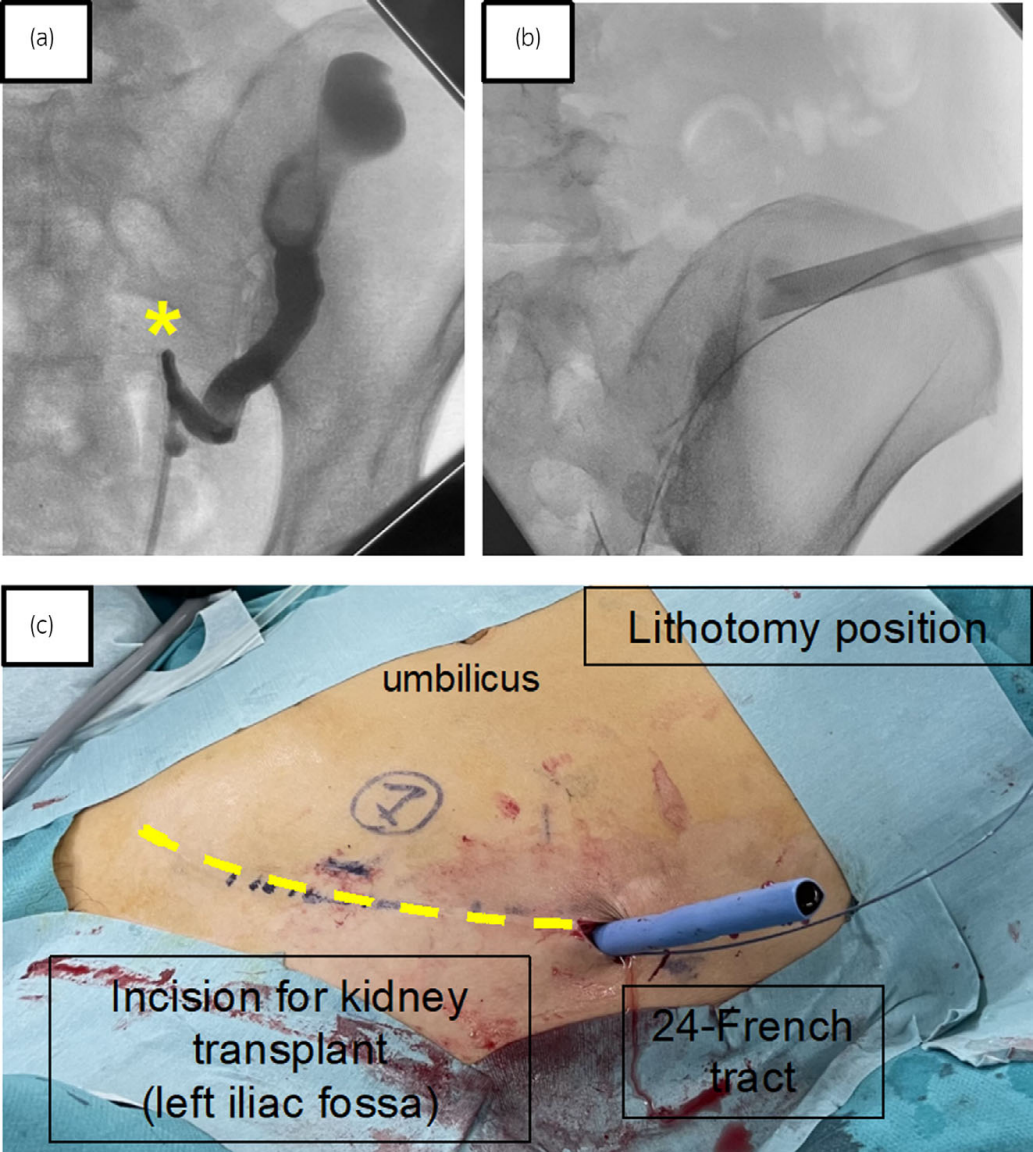
Retrograde pyelography findings and creation of a percutaneous tract. (a) Intraoperative retrograde pyelography findings. The asterisk (*) indicates the site where considerable bending of the ureter was observed (presumed to be the anastomosis site between the native and transplanted ureters). While contrast flow was observed, ureteral mobility was poor; thus, the passage of a ureteral catheter or ureteral access sheath was not feasible. (b, c) Percutaneous renal transplant puncture was performed, and a 24‐French tract was placed. The puncture was directed toward the upper renal calyx, and an echo‐free space was identified.

**Fig. 3:**
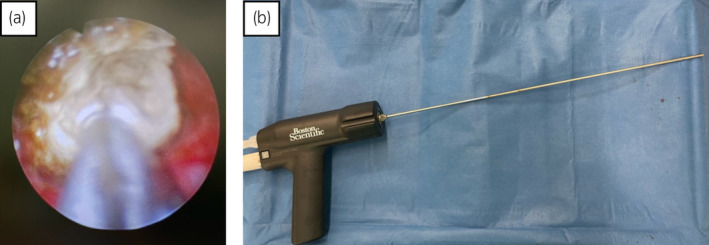
Intraoperative findings and the device used for lithotripsy. (a) Stone fragmentation and aspiration were performed using ultrasonography: intraoperative findings during the initial surgery. (b) Swiss LithoClast^®^ Trilogy lithotripter was used for the second procedure in this case; the lithotripter enables pneumatic and ultrasonic lithotripsy with a single handpiece without the need to exchange probes.

The patient was a 57‐year‐old women with a history of ureteroureterostomy following simultaneous pancreas–kidney transplantation. Fifteen years after transplantation, the patient had gross hematuria, and CT revealed a 23‐mm stone in the transplanted kidney (Fig. 1). The patient was referred from another hospital and had not undergone CT scans regularly. Lithotripsy was performed with the patient under general anesthesia and in the lithotomy position. We initially planned endoscopic combined intrarenal surgery but switched to PCNL using a 6/7.5‐French rigid ureteroscope (Richard Wolf, Germany) because of poor ureter mobility near the anastomosis and significant tortuosity, preventing retrograde access (Fig. 2a). A 24‐French percutaneous tract was created toward the upper pole of the transplanted kidney under ultrasonography and fluoroscopic guidance (Fig. 2b,c). Stone fragmentation was achieved using a combination of pneumatic and ultrasonic lithotripsy (Swiss LithoClast® Master‐J, Switzerland) (Fig. 3a). The first surgery was concluded after placing a nephrostomy catheter because the operation time exceeded 3 h.

**Fig. 4:**
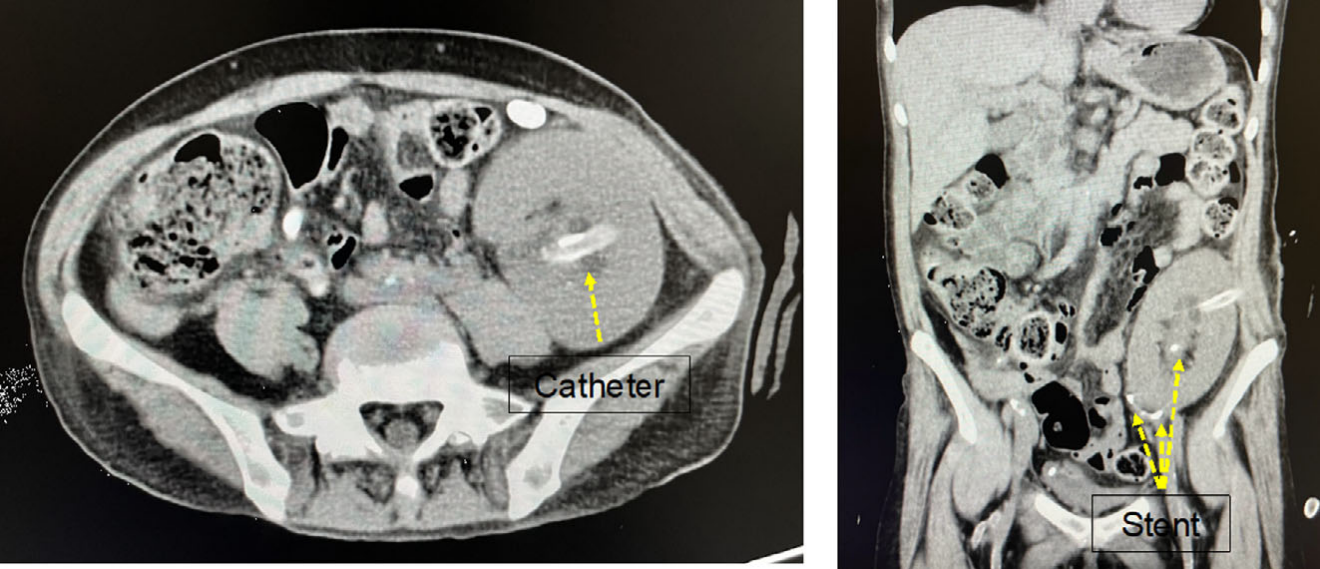
Postoperative CT images. On postoperative Day 1, CT shows that both the nephrostomy catheter and ureteral stent were in place. No residual fragments were observed. The nephrostomy catheter was subsequently removed.

A second PCNL was performed 1 month after the first procedure for residual stones, using Swiss LithoClast® Trilogy, which integrates pneumatic and ultrasonic lithotripsy without the need for probe exchange (Fig. 3b). Smooth stone fragmentation and suctioning resulted in complete stone clearance. Additionally, perhaps because of decreased IRP from the previous nephrostomy tube, a ureteral stent could be placed in the antegrade position during the second surgery. This facilitated the removal of the nephrostomy tube on the first postoperative day after confirming the absence of substantial residual fragments on CT (Fig. 4). Stone analysis revealed calcium oxalate stones. No decline in kidney function or anemia was observed after the two PCNL procedures.

## Discussion

Risk factors for *de novo* kidney allograft stone formation include a prior history of kidney stones, longer dialysis vintage, gout, and hypertension.^2^ It is essential to establish perioperative management and surgical strategies, given that kidney transplant, solitary kidney, and chronic kidney disease under immunosuppression increase susceptibility to infections. Similar to general endoscopic lithotripsy, other essential considerations are appropriate antibiotic therapy based on preoperative urine culture results and management of IRP during surgery.

When the new orifice is located on the anterior wall or the dome of the bladder, there is a tendency for difficulty in accessing the ureteral orifice, which should be considered during ureterovesical anastomosis in kidney transplantation. Additionally, if the ureter is too long, the risk of ureteral injury during ureteroscopy increases. The transplanted ureter, lacking surrounding soft tissue support, tends to be tortuous, making ureteroscopic insertion challenging. Although TUL alone might have been less invasive, concerns about postoperative pyelonephritis from increased IRP, especially given the large stone size and patient background, led to our decision against it.

There are additional specific considerations for TUL and PCNL. TUL requires both rigid and flexible ureteroscopes. Ureteral mobility and variations can complicate intraoperative visualization during stone fragmentation, demanding technical expertise. Additionally, IRP should be considered when drainage is performed. It is also advisable to consider the temporary placement of a thin catheter, such as a pigtail catheter, only during surgery to lower IRP in the transplanted kidney. In PCNL, percutaneous access to the transplanted kidney involves the use of ultrasonography and fluoroscopy, similar to that in conventional nephrostomies. Although the transplanted kidney is located in the iliac fossa, which facilitates percutaneous access in the lithotomy position, its proximity to the bowel loops may necessitate preoperative assessment using CT.^5^ The lithotomy position is also preferred as it facilitates intraoperative transurethral manipulation if needed. Fibrous tissue surrounding the transplanted kidney considerably increases the hardness of the renal capsule during puncture as well as subsequent dilation when compared with the general case. This is because the reactive tissue surrounding the transplanted kidney forms a dense fibrous capsule.^5^ The increased hardness of the renal capsule due to the fibrous tissue surrounding the transplanted kidney often makes it challenging to leave the tract in place directly after balloon dilation during nephrostomy. Therefore, it is essential to adopt a cautious approach during tract placement, gradually dilating from smaller to larger dilators to reduce bleeding. Ultrasonic lithotripters not only generate longitudinal ultrasonic vibrations to finely fragment stones with high precision but also possess suction capability, making them advantageous for minimizing bleeding and obstruction of the surgical field by fragments, thus facilitating stone retrieval. However, as this device cannot be used with flexible ureteroscopes, its use is limited to the fragmentation of stones within the range observed by a rigid nephroscope. Our second surgery featured a notable device that streamlined the process by integrating pneumatic and ultrasonic lithotripsy into a single handpiece (Swiss LithoClast® Trilogy), eliminating the need to change probes. This innovation can increase the efficiency of stone fragmentation and stone retrieval.^6^ ClearPetra® is an alternative device that can be considered, as it enables the procedure to be performed with a smaller sheath, thus achieving a less invasive approach.^7^ In the present case, complications such as postoperative pyelonephritis, surgery‐related kidney dysfunction, and progression of anemia were not observed. With the surgical approach adopted, we could efficiently fragment and retrieve a large kidney allograft stone ≥20 mm in size, without complications.

## Conclusion

Recognizing the characteristics and considerations of the percutaneous approach in PCNL for kidney allograft stones, we demonstrated safe and effective fragmentation of the same using a pneumatic and ultrasonic lithotripsy device.

## Author contributions

Takafumi Yagisawa: Writing – original draft; writing – review and editing. Tomokazu Shimizu: Review and editing. Ayane Tachiki: Review and editing. Yudai Ishiyama: Review and editing. Tadashi Onohara: Review and editing. Shoichi Iida: Review and editing. Hideki Ishida: Supervision. Toshio Takagi: Supervision.

## Conflict of interest

The authors declare no conflict of interest.

## Informed consent

We obtained informed consent from the patient.

## Registry and the Registration No. of the study/trial

Not applicable.

## Approval of the research protocol by the Institutional Reviewer Board

Not applicable.
